# Assessing the effects of 5-HT_2A_ and 5-HT_5A_ receptor antagonists on DOI-induced head-twitch response in male rats using marker-less deep learning algorithms

**DOI:** 10.1007/s43440-024-00679-1

**Published:** 2024-11-27

**Authors:** Ewelina Cyrano, Piotr Popik

**Affiliations:** Behavioral Neuroscience and Drug Development, Maj Institute of Pharmacology, Polish Academy of Sciences, Smętna 12, Kraków, 31-343 Poland

**Keywords:** Head-twitch, 5-HT2A, 5-HT5A, Psychedelic, Machine learning, Computer vision

## Abstract

**Background:**

Serotonergic psychedelics, which display a high affinity and specificity for 5-HT_2A_ receptors like 2,5-dimethoxy-4-iodoamphetamine (DOI), reliably induce a head-twitch response in rodents characterized by paroxysmal, high-frequency head rotations. Traditionally, this behavior is manually counted by a trained observer. Although automation could simplify and facilitate data collection, current techniques require the surgical implantation of magnetic markers into the rodent’s skull or ear.

**Methods:**

This study aimed to assess the feasibility of a marker-less workflow for detecting head-twitch responses using deep learning algorithms. High-speed videos were analyzed using the DeepLabCut neural network to track head movements, and the Simple Behavioral Analysis (SimBA) toolkit was employed to build models identifying specific head-twitch responses.

**Results:**

In studying DOI (0.3125–2.5 mg/kg) effects, the deep learning algorithm workflow demonstrated a significant correlation with human observations. As expected, the preferential 5-HT_2A_ receptor antagonist ketanserin (0.625 mg/kg) attenuated DOI (1.25 mg/kg)-induced head-twitch responses. In contrast, the 5-HT_5A_ receptor antagonists SB 699,551 (3 and 10 mg/kg), and ASP 5736 (0.01 and 0.03 mg/kg) failed to do so.

**Conclusions:**

Previous drug discrimination studies demonstrated that the 5-HT_5A_ receptor antagonists attenuated the interoceptive cue of a potent hallucinogen LSD, suggesting their anti-hallucinatory effects. Nonetheless, the present results were not surprising and support the head-twitch response as selective for 5-HT_2A_ and not 5-HT_5A_ receptor activation. We conclude that the DeepLabCut and SimBA toolkits offer a high level of objectivity and can accurately and efficiently identify compounds that induce or inhibit head-twitch responses, making them valuable tools for high-throughput research.

## Introduction

Due to their established and potential novel therapeutic effects, serotonergic psychedelics have become a focal point of current psychopharmacological research [1]. Clinical trials have demonstrated promising effects of these compounds in alleviating anxiety and depression [2–4], treating substance abuse [5–8], and managing obsessive-compulsive disorder [9, 10]; for recent reviews, see Hodge et al., [11], Madden et al., [12], and Graziosi et al., [13]. However, to identify novel (non)psychedelics and understand their mechanisms of action, animal models remain essential.

Hallucinogen-like effects in rodents can be detected through direct observation, as psychedelics induce a characteristic head-twitch response (HTR) [14, 15]. Rodents treated with various serotonergic psychedelics exhibit bouts of paroxysmal, high-frequency head rotations; for example, in mice, each individual head-twitch lasts approximately 10 milliseconds. In rats, the response to hallucinogens involves a combination of head movements and full-body shakes, resembling the behavior of dogs shaking off water. For this reason, in rats, this behavior is sometimes referred to as a ‘wet dog shake’ [16]. However, for simplicity, we will use the term ‘head-twitch response’ throughout this paper.

This behavior can be observed and manually counted during the test without requiring specialized instruments. To make the observation more objective, faster and reliable, Halberstadt and Geyer [17]; Halberstadt [18], and more recently Shahar et al., [19], developed electronic assessment techniques that allow for the detection of head-twitch behavior with high sensitivity and specificity. In Halberstadt’s method, anesthetized mice are surgically implanted with a neodymium magnet on their cranium. In contrast, the technique by Shahar et al., [19] avoids surgical implantation and instead uses magnetic ear tags [20]. In both methods, the head movements of mice placed in a container are analyzed using specific software. These magnetic marker techniques have proven useful for studying novel hallucinogens.

**Fig. 1:**
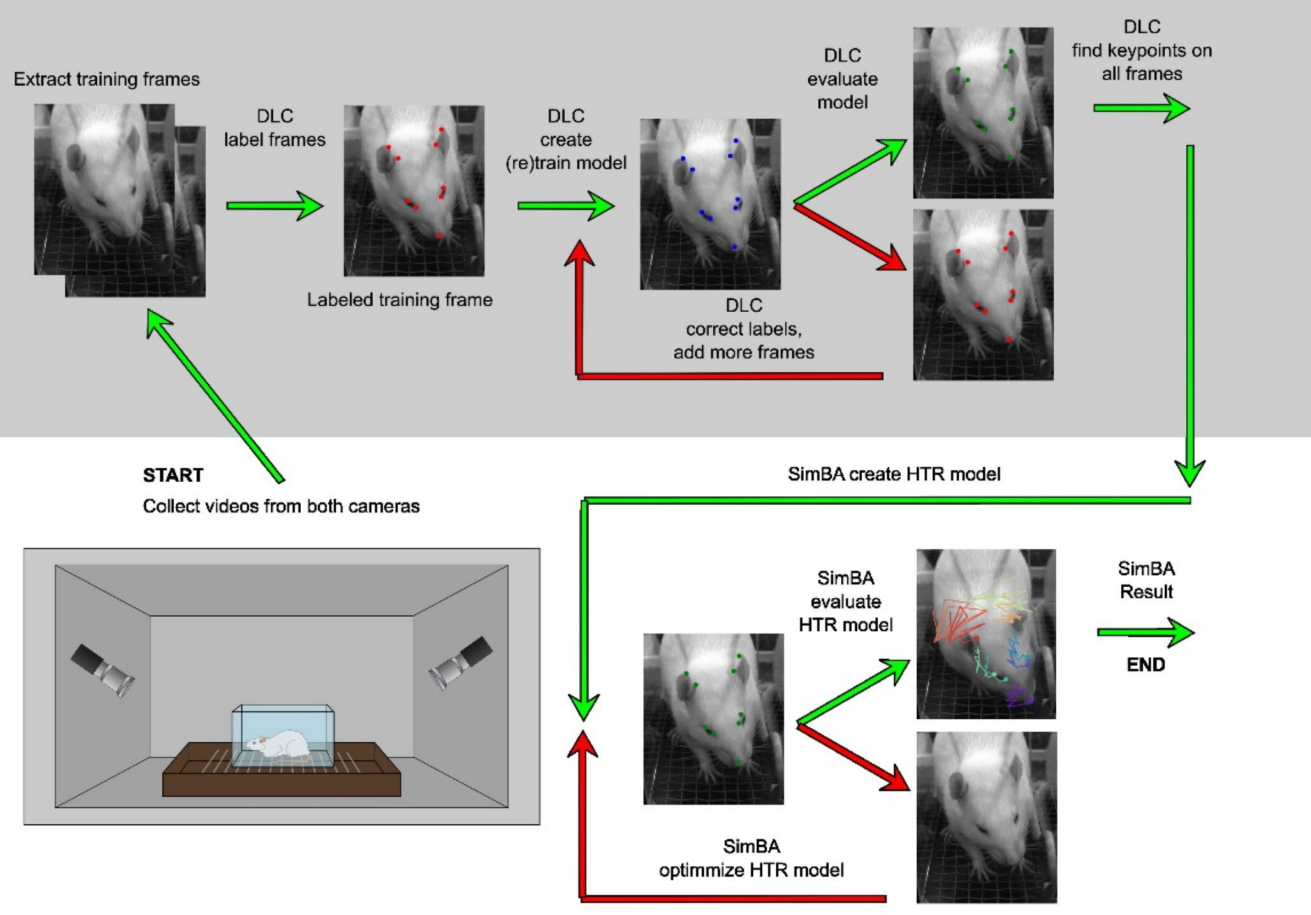
The digital workflow explaining pose estimation training and testing (grayed upper part; DeepLabCut [DLC]) and automatic head-twitch classification (SimBA) algorithms. Bottom left: the setup included a sound-attenuated box with cameras positioned on the sides and a small glass aquarium placed centrally on a wire mesh frame

Recent advances in artificial intelligence, machine learning, and computer vision have enabled the use of marker-less methods for identifying body parts of animals [21]. In this context, the open-source DeepLabCut marker-less pose-estimation toolkit (https://github.com/DeepLabCut) has greatly simplified the analytical workflow [22–24]. DeepLabCut allows for the creation of neural networks (models) that track the movement of animals’ body parts in both time and space. The next step in the analysis involves post-processing with another machine learning software that utilizes the numerical data from DeepLabCut to detect and classify specific behaviors (Fig. 1). In this regard, the Simple Behavioral Analysis (SimBA) toolkit (https://github.com/sgoldenlab/simba) represents a significant advancement in analysis [25]. The primary aim of the present study was to evaluate the feasibility of using freely available, non-invasive, markerless techniques for detecting head-twitch responses in rats.

Converging lines of evidence suggest that serotonin 2 A (5-HT_2A_) receptors play a critical role in the head-twitch response to psychedelic administration. The involvement of 5-HT_2A_ receptors in the effects of serotonergic psychedelics was first demonstrated by Leysen et al., [26], Glennon et al., [27], and Wing et al., [28]. The activation of 5-HT_2A_ receptors as a key mechanism of action for psychedelics is further supported by human studies using pharmacological antagonism [29–32].

2,5-Dimethoxy-4-iodoamphetamine (DOI) is a pharmacological tool commonly used in research to study psychedelics, as it has been shown to reliably induce head-twitch responses in rats [33–37]. Given that this specific 5-HT_2A_ receptor agonist produces also reliable hallucinogenic effects in humans [38], the ability of DOI to induce the head-twitch response demonstrates the high predictive validity of this behavioral model. It is also known that the head-twitch response is one of the few behaviors that can reliably distinguish hallucinogenic from non-hallucinogenic 5-HT_2A_ receptor agonists. For example, while D-lysergic acid diethylamide (LSD) induces head-twitch responses in both mice and rats, the non-hallucinogenic 5-HT_2A_ receptor agonists lisuride [39] and 2-Br-LSD [40] do not elicit this behavior.

Recent data from this laboratory have also implicated the involvement of receptors beyond the ‘classic’ 5-HT_2A_ receptors in the interoceptive effects of the hallucinogen LSD. In rats trained to discriminate LSD from a vehicle, LSD produced dose-dependent increases in responses, which were attenuated by the 5-HT_2A_ receptor antagonist ketanserin [41]. Interestingly, we observed a similar reduction in the LSD cue when 5-HT_5A_ receptor antagonists SB 699551 and ASP 5736 were used. Although the ‘hallucinogenic’ nature of the interoceptive cue produced by LSD in rats remains unclear, this finding suggests that 5-HT_5A_ receptor antagonists may inhibit the subjective effects of LSD [41].

Building on this research, we aimed to investigate whether the same 5-HT_2A_ and 5-HT_5A_ receptor antagonists (ketanserin, SB 699551, and ASP 5736) would affect the head-twitch response following administration of the potent psychedelic DOI in rats. To achieve this, we employed a marker-less workflow utilizing the DeepLabCut and SimBA deep learning analytical tools.

## Methods

### Drugs

Based on the work of Schreiber et al., [35], the 5-HT_2A_ receptor agonist DOI (2,5-dimethoxy-4-iodoamphetamine; Tocris Bioscience, Bristol, UK) was tested at four doses of 0.3125, 0.625, 1.25, and 2.5 mg/kg. The serotonin receptor antagonist ketanserin, synthesized at the Department of Medicinal Chemistry of the Maj Institute of Pharmacology, was administered at a single dose of 0.625 mg/kg [35]. The 5-HT_5A_ receptor antagonist SB 699,551 (N-[2-(Dimethylamino)ethyl]-N-[[4’-[[(2-phenylethyl)amino]methyl][1,1’-biphenyl]-4-yl]methyl]cyclopentanepropanamide dihydrochloride; Tocris Bioscience, Bristol, UK) was used at doses of 3 and 10 mg/kg, while ASP 5736 (N-(diaminomethylene)-1-(3,5-difluoropyridin-4-yl)-4-fluoroisoquinoline-7-carboxamide (2E)-but-2-enedioate), synthesized at the Department of Medicinal Chemistry, was administered at doses of 0.01 and 0.03 mg/kg. The doses of 5-HT_5A_ receptor antagonists were selected based on an earlier report from this laboratory [41]. Physiological saline was used as a placebo. All compounds were administered intraperitoneally (IP) in a volume of 1 ml/kg. DOI was injected immediately before the test, while ketanserin, SB 699,551, and ASP 5736 were administered 30 min before the test. All compounds were dissolved in sterile water immediately before use and administered by an observer blinded to the treatment conditions.

### Animals

Experiments were conducted on 24 male Wistar rats (Charles River, Germany) weighing approximately 225–250 g upon arrival. The animals were housed in groups of four per cage in standard laboratory conditions, with controlled environmental settings: room temperature of 21 ± 2 °C, humidity levels of 40–50%, and a 12-hour light/dark cycle (lights on at 06:00). Food and water were provided *ad libitum*. The animals were acclimatized for approximately three weeks before the start of the experiments, during which time they were handled and familiarized with the experimental apparatus.

All animals were cared for, and experiments were performed in accordance with the European Guidelines for animal welfare (2010/63/EU). The experimental procedures were approved by the II Local Ethics Committee for Animal Experiments at the Maj Institute of Pharmacology, Polish Academy of Sciences, Kraków, Poland (ethical approval 198/2024).

### Video recording

The experiment was conducted inside a custom-built, sound-attenuating box (dimensions: 85 × 45 × 45 cm) equipped with a fan for ventilation and an LED strip providing 745 lx of light at the base, suitable for high-speed video recording. Two digital cameras (Basler acA1440-220 micro m) were positioned at 45° angles on the inner walls of the box. At the center of the box, a glass aquarium (17 × 10.5 × 9 cm) was placed ‘upside-down’ on a wire mesh frame to allow airflow from below. The camera setup ensured that the rat’s head remained visible in either the left or right camera throughout the whole recording, because the animals could move freely from side to side (Fig. 1, bottom).

Simultaneous video recording from both cameras was synchronized using the Arduino platform (Arduino Uno Rev3), programmed with a custom Campy script (https://github.com/ksseverson57/campy). The Arduino device precisely triggered both cameras’ functions. The cameras recorded H.265 encoded, 1440 × 1088 px MP4 files at 60 fps with a variable bitrate.

### Procedure

Tests were conducted during the light phase of the light/dark cycle. On the test day, rats were weighed and transferred to the experimental room, where they were allowed to acclimate for at least 30 min before the experiment began. Following drug administration, the animals were gently placed into the aquarium. Their behaviors were digitally video recorded for 45 min. After each session, the aquarium and floor were cleaned with soapy water and thoroughly dried.

### Experiment design

In Experiment 1, we investigated the effect of DOI’s various doses on the head-twitch response, while in Experiment 2, we examined the effects of pretreatment with 5-HT_2A_ and 5-HT_5A_ receptor antagonists on DOI-induced behavior.

### Sample size estimation

The sample size analysis, conducted using the G*Power program, was based on the results of a published experiment by Rivera-Garcia et al., [37], as it involved the same species (male rats), compounds (DOI and ketanserin), and intraperitoneal administration route. The effect size, based on the means of the four study groups and pooled standard deviation assuming moderate trait variability in the population, was Cohen’s f = 0.924. Given a significance level of *P* < 0.05 and a test power of 0.95, the analysis suggested that eight animals per group were required.

### Randomization

In both experiments, treatments were administered in a random order to prevent carryover effects and to ensure the random selection of treatment conditions, thereby avoiding the influence of the day, or time of day factors on treatment response. No more than two tests per animal per week were conducted to prevent possible tolerance or sensitization to DOI’s effects. Randomization was performed using the R language scripts with the ‘crossdes’ package.

### Digital workflow

The digital workflow is shown on Fig. 1.

### Training pose estimation model using DeepLabCut

The head movements of the rats were automatically tracked using the ‘single animal’ class in DeepLabCut software version 2.3.5 [22]. A total of 670 training frames were extracted from eight randomly selected experimental videos, with 331 frames from the left camera and 339 frames from the right camera. Most frames were automatically selected using the k-means algorithm, and additional frames that varied from the majority were manually added to the training set.

**Fig. 2:**
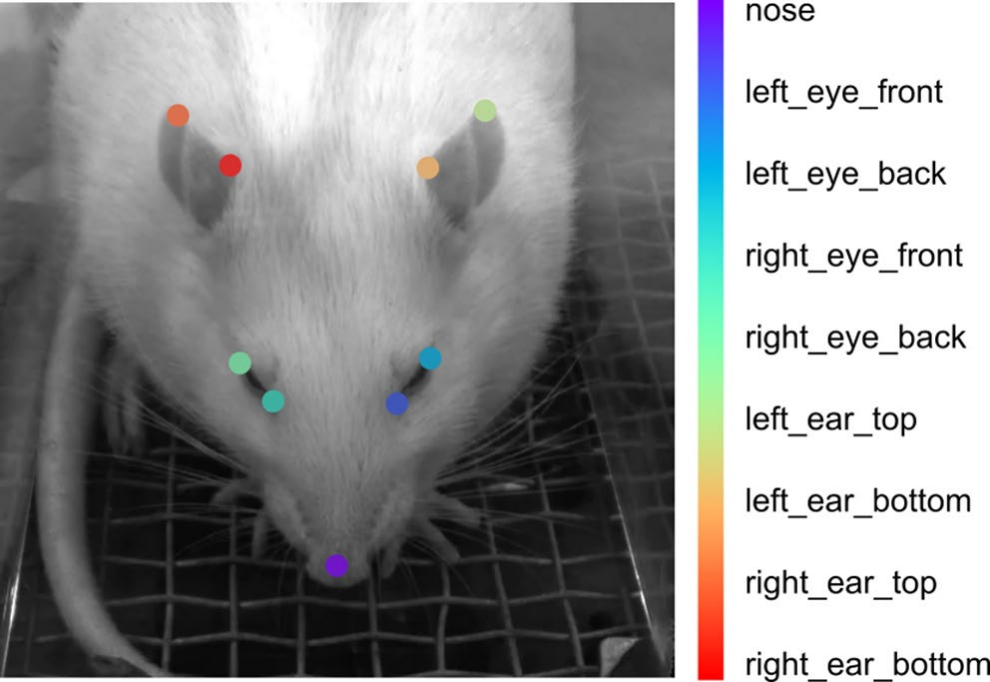
Pose estimation key points used for DeepLabCut model training

The training dataset was created by manually labeling nine key points on the rat’s head in each extracted frame, including the nose, left eye front, left eye back, right eye front, right eye back, left ear bottom, left ear top, right ear bottom, and right ear top (Fig. 2). Only points that were clearly visible in the frame were labeled. The labeling was performed using DLC’s ‘napari’ plug-in, version 0.4.17rc8.

The neural network was trained on Windows 11 Asus Prime Z790-A WIFI PC, equipped with an Intel Core i7-12300 K 3.40 GHz processor, 32 GB of RAM, and a NVIDIA RTX 4090 graphics card. The training utilized the ResNet50 architecture, with 500,000 iterations and imgaug augmentation. The best-performing neural network was then used to analyze the rat’s head movements in each video. The resulting numerical data (CSV files), representing the position of each body part in space and time, were subsequently used to train SimBA’s head-twitch classifier.

### Automatic head-twitch classification using SimBA

Head-twitch episodes were classified using the Simple Behavioral Analysis (SimBA) toolkit (version 1.65.6) [25]. Videos, along with their corresponding tracking data, were imported into the SimBA project to train the head-twitch machine model. The tracking data were imported with the following settings: file type - CSV, interpolation - body parts quadratic, and smoothing - none. The outlier correction step in SimBA was skipped to avoid artificially interpolating body parts during rapid head movements. In addition to the default feature extraction, we calculated frame-by-frame body-part movements as described in the tutorial found at https://github.com/sgoldenlab/simba/blob/master/docs/feature_subsets.md.

Four of the imported videos were manually labeled by a human annotator to mark head-twitch responses. A total of 432,000 frames (equivalent to 2 h of recordings at 60 fps) were inspected, and 271 frames containing head-twitch episodes were annotated. This dataset was used to train the random forest models in SimBA with the following hyperparameters: 2000 random forest estimators, 1 minimum sample leaf, RF criterion = entropy, RF max features = sqrt, test size = 0.2, and oversample parameters ranging from 0 to 16 using the SMOTE oversample setting or undersample parameters (0–16). The classifier with the highest F1 score and best performance upon direct video inspection at slow speed was selected to detect head-twitch episodes in all videos. The minimum bout length was set to 48 ms (equivalent to 3 frames at 60 fps) to capture even the shortest events.

### Statistics

The data were checked for normality using the Anderson-Darling, D’Agostino & Pearson, Shapiro-Wilk, and Kolmogorov-Smirnov tests. As the data did not meet the criteria for normal distribution, they were analyzed using the Kruskal-Wallis ANOVA, followed by Dunn’s multiple comparison tests. Nonlinear regression was employed to determine the ED_50_ of DOI, while simple linear regression was used to compare results from human and digital observations. All statistical analyses were conducted using GraphPad Prism version 9.5.1.

## Results

### Digital workflow evaluation

The DeepLabCut network demonstrated strong performance, with training and test errors of 2.73 and 4.01 pixels, respectively, on 1440 × 1088 px recordings. Applying a pcutoff of 0.6 further reduced the training and test errors to 2.6 and 3.37 pixels, respectively.

**Fig. 3:**
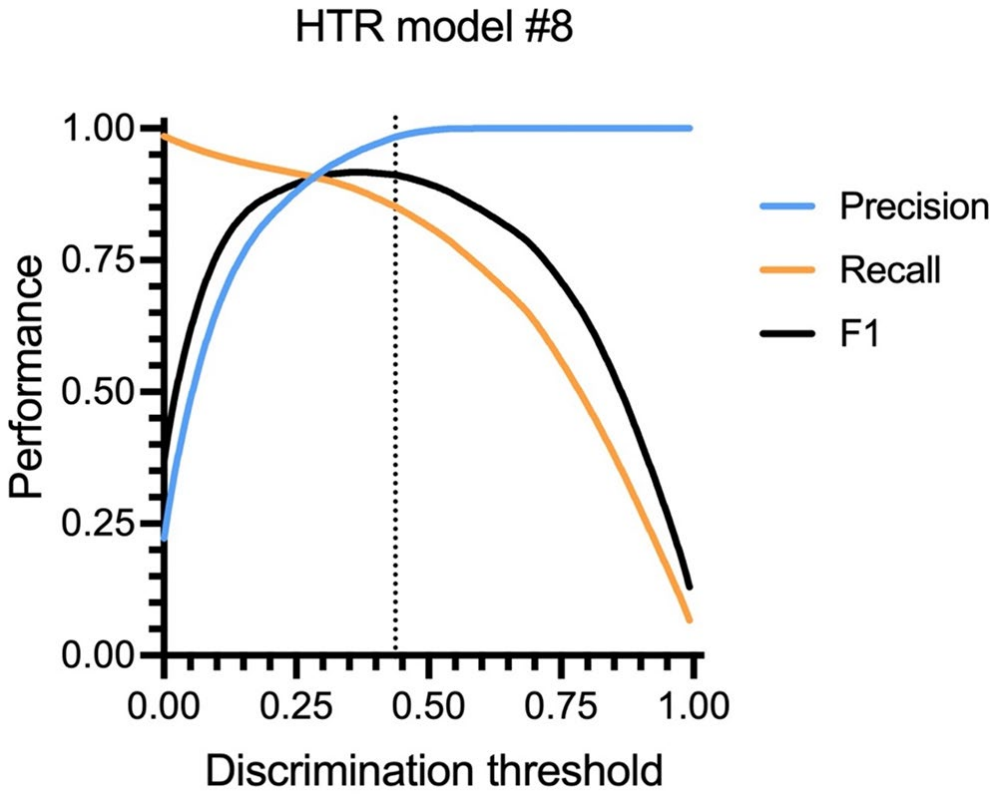
Validation of the performance of the SimBA head-twitch model (classifier) #8. Discrimination threshold values were used to determine the presence of the behavior. Precision (reflecting the impact of false positives), recall (reflecting the impact of false negatives), and F1 scores (the weighted average of precision and recall) are displayed across various thresholds

SimBA’s head-twitch classifiers were evaluated using precision, recall, and F1 scores at various discrimination thresholds, which represent cutoff values for determining the presence of a behavior. The best-performing model #8, achieved the following performance scores: precision 1.0, recall 0.877, and an F1 score of 0.934 at a discrimination threshold of 0.437 (Fig. 3).

**Fig. 4:**
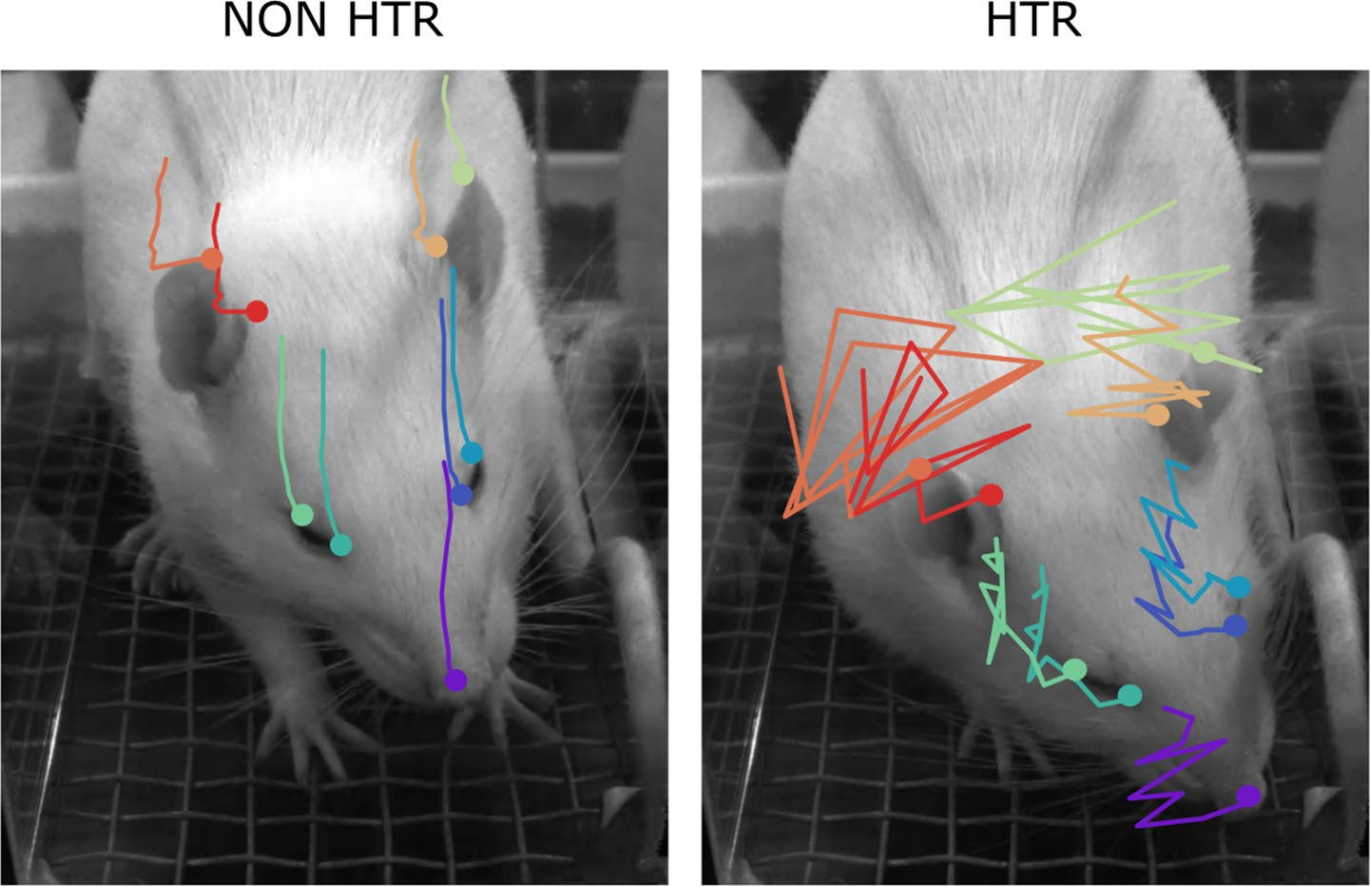
Reconstruction of the head movement sequences without and with a head-twitch episode in the rat, showing the movement of his tracking points. The head position was sampled every 16.67 ms

A single head-twitch episode consisted of 5–11 head movements (Fig. 4), with each movement lasting approximately 11 ms, consistent with observations by Halberstadt and Geyer [17] and Halberstadt [18] in mice.

### Effects of DOI on head-twitch response in rats

**Fig. 5:**
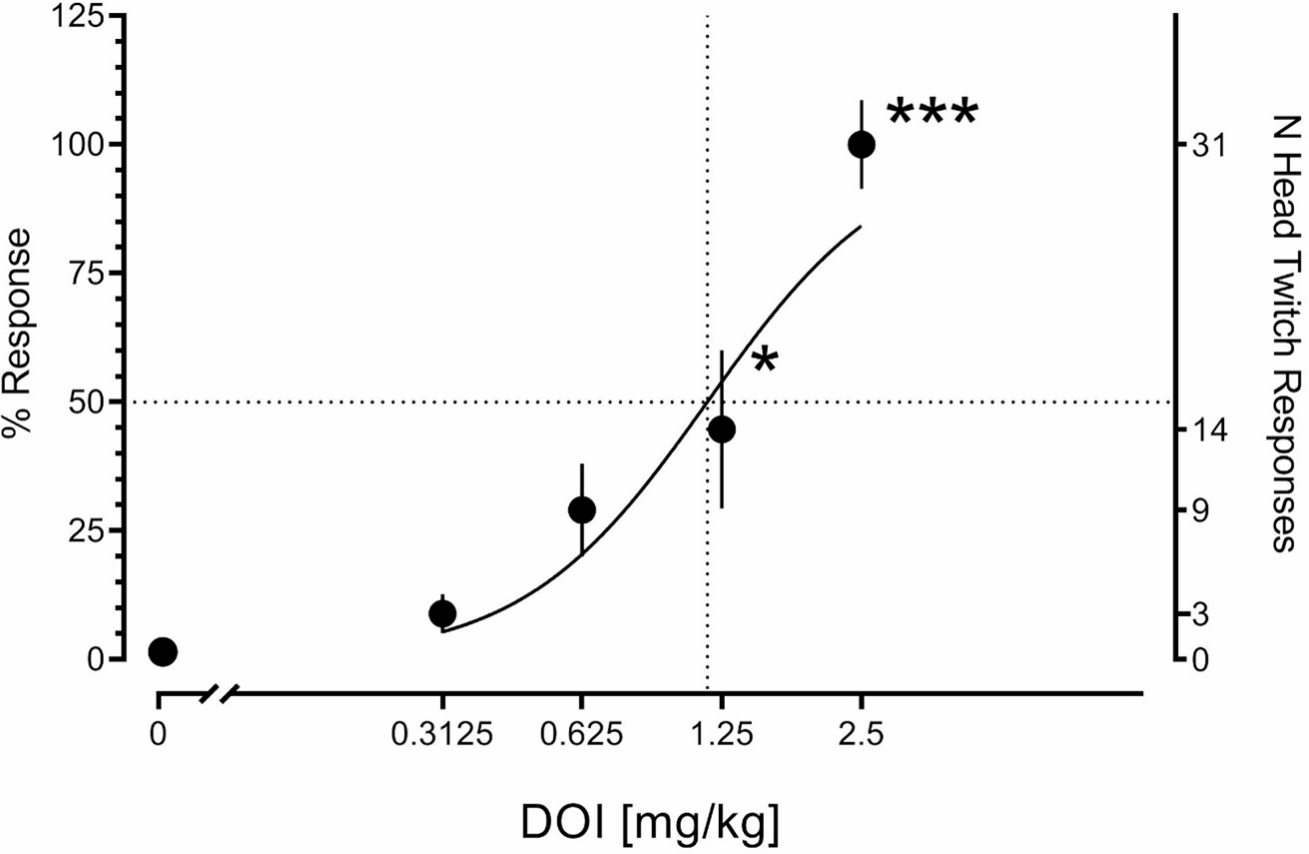
Effect of DOI on head-twitch response in rats. The figure presents the mean ± SEM number of responses detected by DeepLabCut and SimBA digital workflow over the 45-minute test sessions, expressed as the percentage of the maximum. The right axis shows the actual numbers. *Symbols*: *p < 0.05, ***p < 0.001, a significant difference from the vehicle group (Dunn’s multiple comparison test following Kruskal-Wallis ANOVA). The number of measurements per dose tested was 4–7

Following DOI administration, the animals displayed a dose-related increase in the head-twitch response (Fig. 5). Kruskal-Wallis ANOVA demonstrated a significant dose factor (KW (5) = 17.04; *p* < 0.01) with the doses of 1.25 and 2.5 being significantly different from vehicle at *p* < 0.05 and *p* < 0.001, respectively. Non-linear regression demonstrated ED_50_ of 1.163 mg/kg (CI: 0.8370–1.560) with a Hill slope of 2.187.

### A comparison of human and computer wet-dog shake detections

**Fig. 6:**
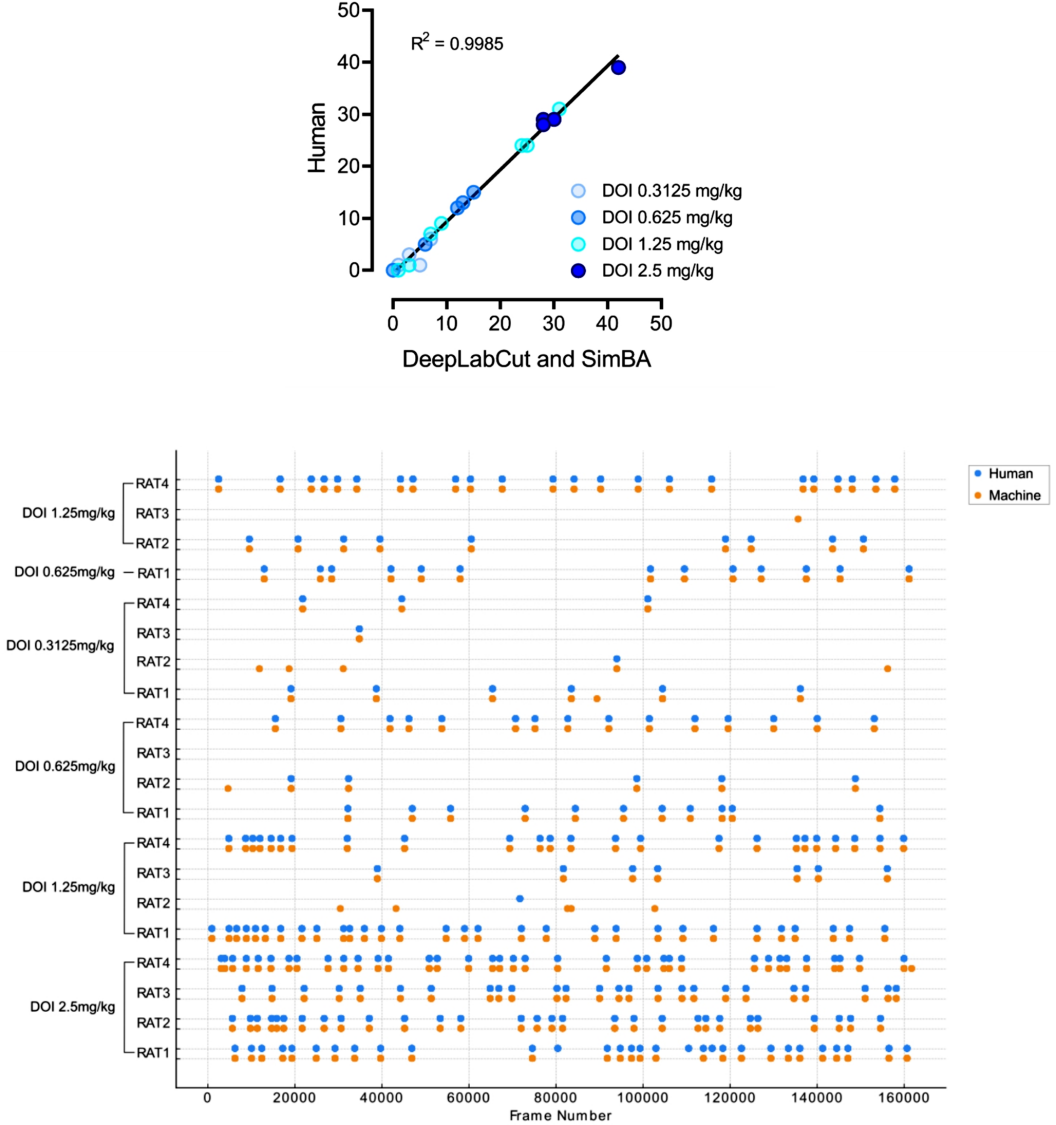
**Upper panel**: A high correlation between head-twitch counts in the videos analyzed by the experienced researcher (Human) and a digital workflow (DeepLabCut and SimBA toolkits). Each point represents the number of head-twitch responses observed on a separate video. The **lower panel** shows the same data as a sequence of observed behaviors for each rat at different DOI doses, analyzed by the machine (orange dots) and the human (blue dots). Each row corresponds to a specific animal and dose

Based on 20 observations of animals treated with DOI, we compared the performance of a trained observer with the digital workflow in detecting head-twitch responses (Fig. 6). The results revealed high similarity between human and machine results with r^2^ = 0.9985 (CI: 0.9518 to 1.045; *p* < 0.0001).

### Effects of 5-HT2A and 5-HT5A receptor antagonists on head-twitch response produced by DOI

**Fig. 7:**
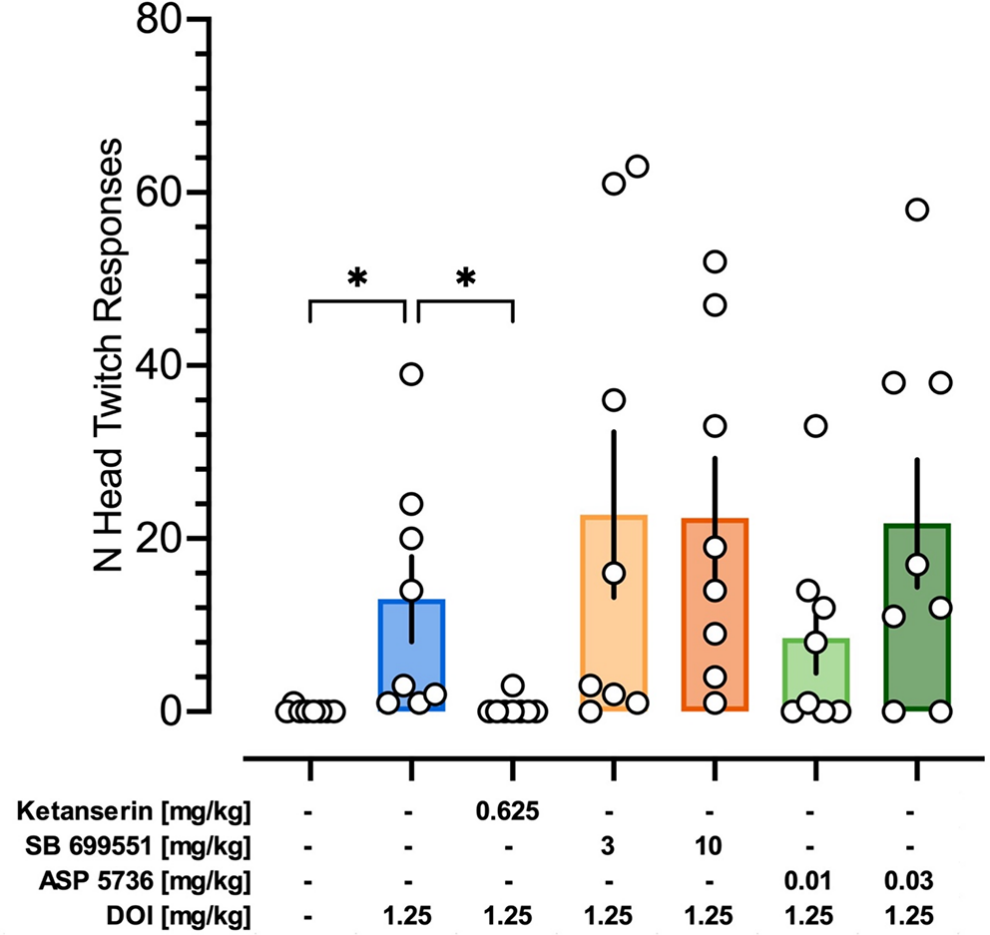
Effects of serotonin receptors’ antagonists on DOI-induced HTR responses in rats. Data are shown as the median ± interquartile range number of responses detected by DeepLabCut and SimBA digital workflow over the 45-minute test session following pretreatment with a vehicle, 5-HT_2A_ receptor antagonist ketanserin, and 5-HT_5A_ receptor antagonists SB 699,551 and ASP 5736 in DOI (1.25 mg/kg)-treated rats. *Symbols*: *p < 0.05, a significant difference from the DOI 1.25 mg/kg group (Dunn’s multiple comparison test following Kruskal-Wallis ANOVA). For each measurement, the *N* = 8

Kruskal-Wallis ANOVA demonstrated significant variability among the groups tested (KW (7) = 25.97; *p* < 0.001). While DOI at 1.25 mg/kg increased the head shake response (*p* = 0.0204) and the 5-HT_2A_ receptor antagonist ketanserin attenuated DOI-induced head-twitch response (*p* = 0.0282), the 5-HT_5A_ receptor antagonists SB 699,551 and ASP 5736 did not affect DOI’s action (Fig. 7).

## Discussion

The present study demonstrates the utility of a non-invasive, markerless digital video workflow in detecting head-twitch responses in rats following the administration of the tool psychedelic compound DOI.Emerging digital techniques, including machine learning toolkits, allow for rapid and objective measurements of animal behavior; for reviews on DeepLabCut, see [42, 43]. The SimBA open-source package, with its user-friendly graphical interface, has already been employed to characterize behaviors such as mouse and rat aggression [25], rat maternal behavior [44], the effects of ketamine on rat social behavior [45], and whether the onset of the dark phase of the light/dark cycle affects rats social activity [46].

We observed a remarkable similarity between the annotations made by an experienced researcher and the results from the digital workflow in detecting the rat head-twitch response. The setup was cost-effective, utilizing a relatively fast computer with a decent graphics card, two digital cameras, and an affordable Arduino card. Both DeepLabCut and SimBA are open-source, free, and ready-to-use Python toolkits that do not require extensive knowledge of deep learning or artificial intelligence algorithms. They are also supported by helpful authors and online communities. Importantly, this method eliminates the need for surgical implantation of magnets in the rodent’s skull or ear. Overall, DeepLabCut and SimBA toolkits provide nearly instantaneous, objective, and precise results with reasonable involvement from the experimenter, making them well-suited for high-throughput research.

We used male rats only because there are either no differences observed, or the existing reports indicate mixed effects regarding sex differences in the HTR response to psychedelics administration (see Shadani et al., [47] for a recent review). Nonetheless the present results align with numerous previous studies demonstrating the head-twitch response in rats following DOI administration [33–37]; see Canal and Morgan [15] for an extensive review. The high correlation between human and computer-based detections supports the use of a digital workflow for studying the effects of serotonin receptor antagonists on DOI’s actions. The specificity of DOI’s effect was confirmed by its attenuation with the preferential 5-HT_2A_ receptor antagonist, ketanserin, consistent with earlier rat studies [33, 35–37] and human observations [29–32], which demonstrate the inhibition of psychedelic effects by this medication.

However, we failed to detect an inhibitory action of the 5-HT_5A_ receptor antagonists SB 699551 and ASP 5736 on DOI-induced head-twitch responses. This failure could be due to the fact that this assay primarily relies on the activation of 5-HT_2A_ receptors, to which SB 699551 and ASP 5736 do not demonstrate significant affinity. Specifically, SB 699551 compound’s affinity at 5-HT_2A_ receptors is as low as ~ 6.1 (pK_i_) but the compound displays high-affinity (pK_i_ of 8.5) at 5-HT_5A_ receptor with at least 30-fold selectivity (except for the serotonin transporter) for the human 5-HT_5A_ receptor over several other 5-HT and other CNS receptors [48]. Similarly, ASP5736 exhibits no affinity at 5HT_2A_ receptor, but high (3.6 nM, K_i_) affinity) [49] and selectivity for human 5-HT_5A_ receptors (with the exception of 5-HT_2C_ and 5-H_T7_ receptors) over 51 receptors, 5 ion channels, 3 enzymes, and 3 transporters [49, 50].

5-HT_5A_ receptor antagonists exhibit effects commonly associated with antipsychotic medications. For instance, Jongen-Relo et al., [51] reported that the 5-HT_5A_ receptor antagonists A763079 and A843277 antagonized methamphetamine- and MK-801-induced hyperactivity. Additionally, these authors found that A763079 and A843277 demonstrated other antipsychotic-like effects, such as enhancing prepulse inhibition of the acoustic startle response in DBA/2 mice.

However, while SB 699,551 demonstrated antipsychotic-like activity in ameliorating ketamine-induced social withdrawal in the social choice test [52] and ASP5736 reduced positive-like symptoms and cognitive impairment in animal models of schizophrenia [49], these effects were not necessarily linked to 5-HT_2A_ receptor inhibition. From this perspective, it is not surprising that while these compounds inhibited the subjective cue produced by hallucinogenic LSD in the drug-discrimination study [41], they did not affect DOI-induced head-twitch response, underlying this assay’s specificity for 5-HT_2A_ receptor activation and inhibition.

In summary, we conclude that the novel, non-invasive marker-less digital technique provides a high level of objectivity and accuracy, enabling the rapid detection of compounds that induce or inhibit the head-twitch response. However, the present study does not support the involvement of 5-HT_5A_ receptor antagonists in this phenomenon, though this does not necessarily negate their potential utility as anti-hallucinogenic or antipsychotic agents.

## Data Availability

Data that support the findings of this study are available at 10.17605/OSF.IO/JUE85.
